# Supplementary material for: Tead1a Initiates Transcriptional Priming Through the TEAD1a/YAP‐Notch1‐Spi1/Cebpα Axis to Promote Neutrophil Fate
Source: Adv Sci (Weinh). 2025 Aug 28;12(41):e05441. doi: 10.1002/advs.202505441 (PMC12591127; doi:10.1002/advs.202505441)
Supplement: Supplementary file 4 — Supplementary Dataset [file ADVS-12-e05441-s004.zip › 3-6.zip!/3-6/02-6.cluster/cluster16/Enrich/ego_CC.pdf]

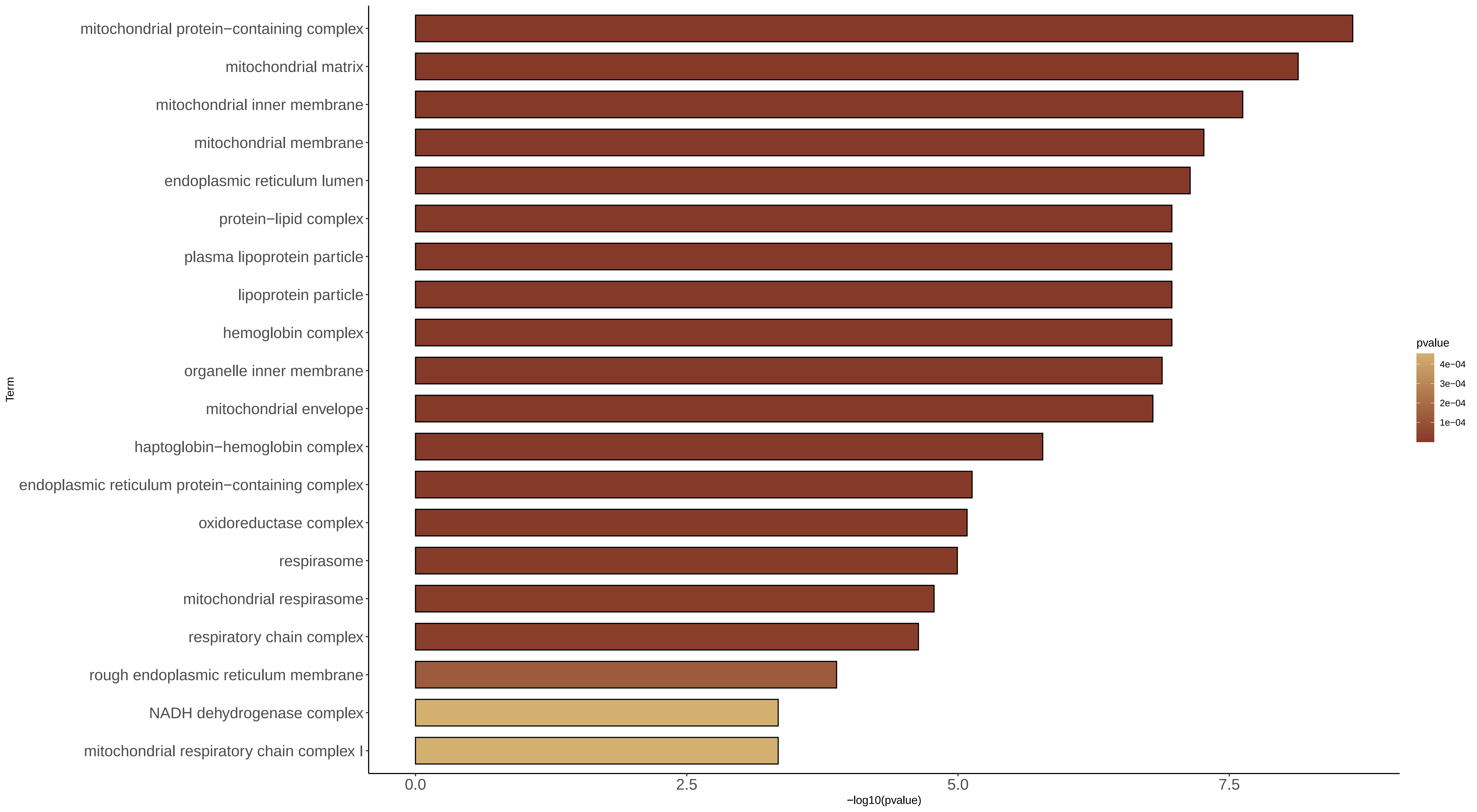
mitochondrial protein−containing complex
mitochondrial matrix
mitochondrial inner membrane
mitochondrial membrane
endoplasmic reticulum lumen
protein−lipid complex
plasma lipoprotein particle
lipoprotein particle
hemoglobin complex
organelle inner membrane
mitochondrial envelope
haptoglobin−hemoglobin complex
endoplasmic reticulum protein−containing complex
oxidoreductase complex
respirasome
mitochondrial respirasome
respiratory chain complex
rough endoplasmic reticulum membrane
NADH dehydrogenase complex
mitochondrial respiratory chain complex I
Term
0.0
2.5
5.0
7.5
−log10(pvalue)
pvalue
4e−04
3e−04
2e−04
1e−04
